# 2,9-Bis(trichloro­meth­yl)-1,10-phenanthroline^1^

**DOI:** 10.1107/S1600536810002035

**Published:** 2010-01-23

**Authors:** Hoong-Kun Fun, Suchada Chantrapromma, Annada C. Maity, Shyamaprosad Goswami

**Affiliations:** a X-ray Crystallography Unit, School of Physics, Universiti Sains Malaysia, 11800 USM, Penang, Malaysia; b Crystal Materials Research Unit, Department of Chemistry, Faculty of Science, Prince of Songkla University, Hat-Yai, Songkhla 90112, Thailand; c Department of Chemistry, Bengal Engineering and Science University, Shibpur, Howrah 711 103, India

## Abstract

The asymmetric unit of the title compound, C_14_H_6_Cl_6_N_2_, contains two crystallographically independent mol­ecules, each of which is slightly twisted from planarity. The dihedral angles between the central ring and the two outer rings are 3.81 (7) and 4.30 (7)° in one mol­ecule, and 4.13 (8) and 4.10 (7)° in the other. In the crystal structure, mol­ecules are inter­linked by C—Cl⋯Cl inter­actions into sheets parallel to the *ac* plane. These sheets are stacked along the *b* axis in such a way that the mol­ecules are anti­parallel; they are further connected into a supra­molecular network. There are no classical hydrogen bonds. C⋯Cl [3.637 (2) Å], Cl⋯Cl [3.5639 (5)–3.6807 (8) Å] and Cl⋯N [3.3802 (15)–3.4093 (15) Å] short contacts and π–π inter­actions, with centroid–centroid distances in the range 3.5868 (9)–3.7844 (9) Å, are observed.

## Related literature

For reference bond-length data, see: Allen *et al.* (1987 ▶). For background to and applications of 1,10-phenanthroline derivatives, see: Armaroli *et al.* 1992 ▶); Beer *et al.* (1993 ▶); Emmerling *et al.* (2007 ▶); Goswami *et al.* (2007 ▶); Wesselinova *et al.* (2009 ▶). For the stability of the temperature controller used in the data collection, see: Cosier & Glazer (1986 ▶).
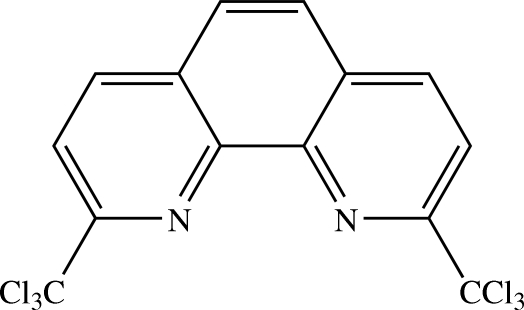


## Experimental

### 

#### Crystal data

C_14_H_6_Cl_6_N_2_
*M* *_r_* = 414.91
Monoclinic, 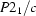

*a* = 24.3001 (6) Å
*b* = 6.8825 (2) Å
*c* = 20.3461 (5) Å
β = 114.689 (1)°
*V* = 3091.74 (14) Å^3^
*Z* = 8
Mo *K*α radiation
μ = 1.11 mm^−1^
*T* = 100 K
0.59 × 0.36 × 0.10 mm

#### Data collection

Bruker APEXII CCD area-detector diffractometer
Absorption correction: multi-scan (*SADABS*; Bruker, 2005 ▶) *T*_min_ = 0.561, *T*_max_ = 0.898
63570 measured reflections
13520 independent reflections
9474 reflections with *I* > 2σ(*I*)
*R*_int_ = 0.054

#### Refinement

*R*[*F* ^2^ > 2σ(*F* ^2^)] = 0.042
*wR*(*F* ^2^) = 0.105
*S* = 1.06
13520 reflections
397 parameters
H-atom parameters constrained
Δρ_max_ = 0.60 e Å^−3^
Δρ_min_ = −0.54 e Å^−3^

### 

Data collection: *APEX2* (Bruker, 2005 ▶); cell refinement: *SAINT* (Bruker, 2005 ▶); data reduction: *SAINT*; program(s) used to solve structure: *SHELXTL* (Sheldrick, 2008 ▶); program(s) used to refine structure: *SHELXTL*; molecular graphics: *SHELXTL*; software used to prepare material for publication: *SHELXTL* and *PLATON* (Spek, 2009 ▶).

## Supplementary Material

Crystal structure: contains datablocks global, I. DOI: 10.1107/S1600536810002035/wn2373sup1.cif

Structure factors: contains datablocks I. DOI: 10.1107/S1600536810002035/wn2373Isup2.hkl

Additional supplementary materials:  crystallographic information; 3D view; checkCIF report

## Figures and Tables

**Fig. 1.**
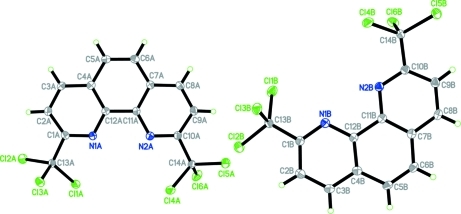
The molecular structure of the asymmetric unit of the title compound, showing 50% probability displacement ellipsoids and the atom-numbering scheme. Hydrogen atoms are shown as spheres of arbitrary radius.

**Fig. 2.**
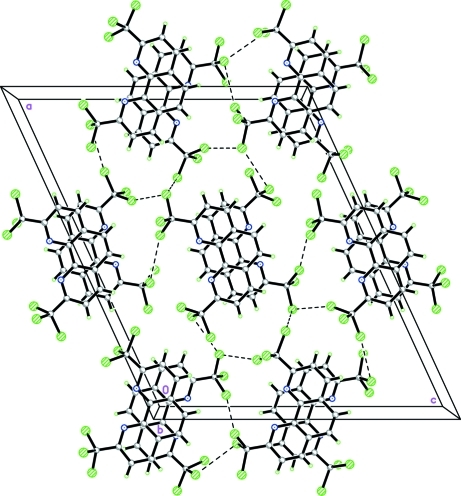
The crystal packing of the title compound,viewed along the b axis. Cl···Cl contacts are shown as dashed lines.

## supplementary crystallographic information

### Comment

Trichloromethyl-substituted heterocyclic compounds are of great importance due to their broad spectrum of biological activities. 2,9-Bis(trichloromethyl)-1,10-phenanthroline is used as a potentially robust ligand for metal oxidation catalysts (Beer *et al.*, 1993). 1,10-phenanthroline derivatives also show antitumor (Wesselinova *et al.*, 2009) as well as luminescence properties (Armaroli *et al.*, 1992). Recently a series of trichloromethyl-substituted heterocyclic compounds has been synthesized (Goswami *et al.*, 2007) in good yield using *N*-chlorosuccinimide (NCS) and triphenylphosphine (PPh_3_) in carbon tetrachloride. In supramolecular chemistry it is known that the self-association of individual molecules can lead to the formation of highly complex and fascinating supramolecular aggregates if halogen···π interactions contribute to the formation of specific motifs (Emmerling *et al.*, 2007). The title compound was synthesized in order to study its supramolecular structure.

The asymmetric unit (Fig. 1) contains two molecules, *A* and *B*, having slight differences in bond lengths and angles. The 1,10-phenanthroline unit is not strictly planar, with dihedral angles between the central ring and the C1–C4/C12/N1 and C7–C11/N2 rings of 3.81 (7) and 4.30 (7)°, respectively, for molecule *A* [the corresponding values for molecule *B* are 4.13 (8) and 4.10 (7)° ]. In both molecules, *A* and *B*, none of the Cl atoms of the trichloromethyl substitutent is coplanar with the 1,10-phenanthroline ring system. The bond distances adopt normal values (Allen *et al.*, 1987).

In the crystal structure (Fig. 2), non-covalent interactions play a significant role in the three-dimensional supramolecular architecture, in which the molecules are interlinked into sheets parallel to the *ac* plane. These sheets are stacked along the *b* axis in such a way that the molecules are antiparallel. These sheets are further connected into a supramolecular network. There are no classical hydrogen bonds. However, C···Cl [3.637 (2) Å], Cl···Cl [3.5639 (5)–3.6807 (8) Å] and Cl···N [3.3802 (15)–3.4093 (15) Å] short contacts are present. π–π interactions are also observed, with distances of *Cg*_1_···*Cg*_2_^i^ = 3.6610 (9) Å, *Cg*_1_···*Cg*_2_^ii^ = 3.5868 (9) Å, *Cg*_1_···*Cg*_3_^i^ = 3.7331 (10) Å, *Cg*_2_···*Cg*_3_^ii^ = 3.7845 (9) Å, *Cg*_4_···*Cg*_5_^iii^ = 3.5949 (9) Å, *Cg*_4_···*Cg*_5_^iv^ = 3.6404 (9) Å, *Cg*_4_···*Cg*_6_^iii^ = 3.7417 (10) Å and *Cg*_5_···*Cg*_6_^iv^ = 3.7198 (9) Å (symmetry codes: (i) 1 - *x*, 1 - *y*, 1 - *z*; (ii) 1 - *x*, 2 - *y*, 1 - *z*; (iii) 2 - *x*, -*y*, 1 - *z* and (iv) 2 - *x*, 1 - *y*, 1 - *z*). *Cg*_1_, *Cg*_2_, *Cg*_3_, *Cg*_4_, *Cg*_5_ and *Cg*_6_ are the centroids of the rings C1A–C4A/C12A/N1A, C7A–C11A/N2A, C4A–C7A/C11A–C12A, C1B–C4B/C12B/N1B, C7B–C11B/N2B and C4B–C7B/C11B–C12B, respectively.

### Experimental

A mixture of *N*-chlorosuccinimide (500 mg, 4.5 mmol) and triphenylphosphine (500 mg, 4.2 mmol) was moistened with CCl_4_ (60 ml) in a round bottom flask and stirred at room temperature for 25 min. A solution of 2,9-dimethyl-1,10-phenanthroline (1 g, 5.2 mmol) was added to the suspension and the reaction mixture was stirred and heated under reflux for 7 h. The solution was cooled and filtered. The evaporated filtrate was washed with saturated aqueous Na_2_CO_3_ and extracted repeatedly with CHCl_3_. Drying over anhydrous Na_2_SO_4_ was carried out, and the solvent was removed under reduced pressure. The crude product was purified with SiO_2_ chromatography (eluted with 1% ethyl acetate in petroleum ether) to give the title compound as a white crystalline solid. Colorless plate-shaped single crystals suitable for *X*-ray structure determination were recrystalized from CH_2_Cl_2_:hexane (1:10, *v*/*v*) by slow evaporation of the solvent at room temperature over the course of a week.

### Refinement

All H atoms were placed in calculated positions, with C—H = 0.93 Å, *U*_iso_(H) = 1.2*U*_eq_(C).

### Crystal data

**Table:**

C_14_H_6_Cl_6_N_2_	*F*(000) = 1648
*M**_r_* = 414.91	*D*_x_ = 1.783 Mg m^−^^3^
Monoclinic, *P*2_1_/*c*	Mo *K*α radiation, λ = 0.71073 Å
Hall symbol: -P 2ybc	Cell parameters from 13520 reflections
*a* = 24.3001 (6) Å	θ = 0.9–35.0°
*b* = 6.8825 (2) Å	µ = 1.11 mm^−^^1^
*c* = 20.3461 (5) Å	*T* = 100 K
β = 114.689 (1)°	Plate, colorless
*V* = 3091.74 (14) Å^3^	0.59 × 0.36 × 0.10 mm
*Z* = 8	

### Data collection

**Table:**

Bruker APEXII CCD area-detector diffractometer	13520 independent reflections
Radiation source: sealed tube	9474 reflections with *I* > 2σ(*I*)
graphite	*R*_int_ = 0.054
φ and ω scans	θ_max_ = 35.0°, θ_min_ = 0.9°
Absorption correction: multi-scan (*SADABS*; Bruker, 2005)	*h* = −34→39
*T*_min_ = 0.561, *T*_max_ = 0.898	*k* = −11→11
63570 measured reflections	*l* = −32→32

### Refinement

**Table:**

Refinement on *F*^2^	Primary atom site location: structure-invariant direct methods
Least-squares matrix: full	Secondary atom site location: difference Fourier map
*R*[*F*^2^ > 2σ(*F*^2^)] = 0.042	Hydrogen site location: inferred from neighbouring sites
*wR*(*F*^2^) = 0.105	H-atom parameters constrained
*S* = 1.06	*w* = 1/[σ^2^(*F*_o_^2^) + (0.0401*P*)^2^ + 1.5309*P*] where *P* = (*F*_o_^2^ + 2*F*_c_^2^)/3
13520 reflections	(Δ/σ)_max_ = 0.003
397 parameters	Δρ_max_ = 0.60 e Å^−^^3^
0 restraints	Δρ_min_ = −0.54 e Å^−^^3^

### Special details

**Table:**

Experimental. The crystal was placed in the cold stream of an Oxford Cryosystems Cobra open-flow nitrogen cryostat (Cosier & Glazer, 1986) operating at 120.0 (1) K.
Geometry. All e.s.d.'s (except the e.s.d. in the dihedral angle between two l.s. planes) are estimated using the full covariance matrix. The cell e.s.d.'s are taken into account individually in the estimation of e.s.d.'s in distances, angles and torsion angles; correlations between e.s.d.'s in cell parameters are only used when they are defined by crystal symmetry. An approximate (isotropic) treatment of cell e.s.d.'s is used for estimating e.s.d.'s involving l.s. planes.
Refinement. Refinement of *F*^2^ against ALL reflections. The weighted *R*-factor *wR* and goodness of fit *S* are based on *F*^2^, conventional *R*-factors *R* are based on *F*, with *F* set to zero for negative *F*^2^. The threshold expression of *F*^2^ > σ(*F*^2^) is used only for calculating *R*-factors(gt) *etc*. and is not relevant to the choice of reflections for refinement. *R*-factors based on *F*^2^ are statistically about twice as large as those based on *F*, and *R*- factors based on ALL data will be even larger.

### Fractional atomic coordinates and isotropic or equivalent isotropic displacement parameters (Å^2^)

**Table:**

	*x*	*y*	*z*	*U*_iso_*/*U*_eq_	
Cl1A	0.334774 (19)	0.88628 (7)	0.25606 (2)	0.02086 (9)	
Cl2A	0.242550 (19)	0.81155 (7)	0.30569 (2)	0.02246 (9)	
Cl3A	0.301547 (19)	0.49030 (6)	0.26994 (2)	0.01983 (9)	
Cl4A	0.55485 (2)	0.64013 (8)	0.29133 (3)	0.02921 (11)	
Cl5A	0.679947 (19)	0.72747 (7)	0.37576 (2)	0.02217 (9)	
Cl6A	0.59362 (2)	1.03890 (7)	0.31954 (3)	0.02459 (10)	
N1A	0.41730 (6)	0.7423 (2)	0.39518 (8)	0.0142 (3)	
N2A	0.53663 (6)	0.7752 (2)	0.41504 (8)	0.0150 (3)	
C1A	0.36107 (7)	0.7161 (2)	0.38719 (9)	0.0148 (3)	
C2A	0.34427 (8)	0.6758 (3)	0.44406 (9)	0.0170 (3)	
H2AA	0.3041	0.6512	0.4352	0.020*	
C3A	0.38910 (8)	0.6741 (3)	0.51314 (10)	0.0164 (3)	
H3AA	0.3795	0.6497	0.5521	0.020*	
C4A	0.44947 (7)	0.7094 (2)	0.52491 (9)	0.0150 (3)	
C5A	0.49733 (8)	0.7192 (2)	0.59632 (9)	0.0164 (3)	
H5AA	0.4888	0.6984	0.6363	0.020*	
C6A	0.55494 (8)	0.7584 (2)	0.60616 (9)	0.0165 (3)	
H6AA	0.5852	0.7696	0.6528	0.020*	
C7A	0.56969 (7)	0.7830 (2)	0.54558 (9)	0.0145 (3)	
C8A	0.62916 (7)	0.8199 (2)	0.55361 (9)	0.0165 (3)	
H8AA	0.6601	0.8368	0.5995	0.020*	
C9A	0.64155 (8)	0.8310 (3)	0.49404 (10)	0.0170 (3)	
H9AA	0.6805	0.8572	0.4985	0.020*	
C10A	0.59347 (7)	0.8014 (2)	0.42572 (9)	0.0150 (3)	
C11A	0.52411 (7)	0.7680 (2)	0.47387 (9)	0.0138 (3)	
C12A	0.46148 (7)	0.7381 (2)	0.46339 (9)	0.0133 (3)	
C13A	0.31304 (7)	0.7276 (2)	0.30927 (9)	0.0155 (3)	
C14A	0.60462 (7)	0.8011 (3)	0.35693 (9)	0.0161 (3)	
Cl1B	0.83130 (2)	0.36601 (7)	0.57545 (3)	0.02271 (9)	
Cl2B	0.74008 (2)	0.25940 (8)	0.43624 (3)	0.02710 (11)	
Cl3B	0.807802 (19)	−0.03889 (6)	0.53631 (2)	0.02007 (9)	
Cl4B	1.05051 (2)	0.13826 (8)	0.76066 (2)	0.02696 (11)	
Cl5B	1.177123 (19)	0.19488 (7)	0.80031 (2)	0.02159 (9)	
Cl6B	1.09840 (2)	0.52724 (7)	0.77611 (2)	0.02242 (9)	
N1B	0.91644 (6)	0.2259 (2)	0.52083 (8)	0.0149 (3)	
N2B	1.03543 (6)	0.2648 (2)	0.62009 (8)	0.0143 (3)	
C1B	0.86038 (7)	0.1972 (2)	0.47266 (9)	0.0150 (3)	
C2B	0.84362 (8)	0.1635 (3)	0.39875 (9)	0.0175 (3)	
H2BA	0.8037	0.1363	0.3676	0.021*	
C3B	0.88840 (8)	0.1721 (3)	0.37394 (9)	0.0170 (3)	
H3BA	0.8789	0.1524	0.3252	0.020*	
C4B	0.94853 (7)	0.2108 (2)	0.42278 (9)	0.0149 (3)	
C5B	0.99605 (8)	0.2322 (2)	0.39896 (9)	0.0163 (3)	
H5BA	0.9876	0.2163	0.3503	0.020*	
C6B	1.05313 (8)	0.2754 (3)	0.44695 (10)	0.0170 (3)	
H6BA	1.0831	0.2953	0.4305	0.020*	
C7B	1.06805 (7)	0.2908 (2)	0.52255 (9)	0.0148 (3)	
C8B	1.12731 (8)	0.3302 (2)	0.57408 (10)	0.0166 (3)	
H8BA	1.1580	0.3539	0.5591	0.020*	
C9B	1.13989 (8)	0.3336 (3)	0.64646 (10)	0.0171 (3)	
H9BA	1.1786	0.3611	0.6811	0.021*	
C10B	1.09210 (7)	0.2938 (2)	0.66644 (9)	0.0146 (3)	
C11B	1.02291 (7)	0.2647 (2)	0.54862 (9)	0.0139 (3)	
C12B	0.96058 (7)	0.2317 (2)	0.49680 (9)	0.0137 (3)	
C13B	0.81276 (7)	0.1983 (3)	0.50346 (9)	0.0159 (3)	
C14B	1.10337 (7)	0.2871 (3)	0.74605 (9)	0.0159 (3)	

### Atomic displacement parameters (Å^2^)

**Table:**

	*U*^11^	*U*^22^	*U*^33^	*U*^12^	*U*^13^	*U*^23^
Cl1A	0.02068 (19)	0.0238 (2)	0.01618 (18)	−0.00383 (15)	0.00582 (15)	0.00361 (16)
Cl2A	0.01529 (18)	0.0299 (2)	0.0216 (2)	0.00706 (15)	0.00717 (16)	0.00172 (17)
Cl3A	0.01796 (18)	0.01901 (19)	0.0200 (2)	−0.00169 (14)	0.00547 (15)	−0.00399 (15)
Cl4A	0.0277 (2)	0.0436 (3)	0.0195 (2)	−0.0168 (2)	0.01296 (18)	−0.0111 (2)
Cl5A	0.01725 (19)	0.0279 (2)	0.0222 (2)	0.00705 (15)	0.00910 (16)	0.00110 (17)
Cl6A	0.0264 (2)	0.0251 (2)	0.0277 (2)	0.00896 (17)	0.01677 (19)	0.01139 (18)
N1A	0.0136 (6)	0.0136 (6)	0.0153 (6)	0.0006 (5)	0.0059 (5)	0.0001 (5)
N2A	0.0151 (6)	0.0146 (6)	0.0156 (6)	0.0006 (5)	0.0068 (5)	0.0008 (5)
C1A	0.0141 (7)	0.0134 (7)	0.0159 (7)	0.0004 (5)	0.0054 (6)	−0.0016 (6)
C2A	0.0139 (7)	0.0192 (8)	0.0185 (8)	−0.0009 (6)	0.0074 (6)	0.0001 (6)
C3A	0.0185 (8)	0.0171 (8)	0.0165 (7)	0.0004 (6)	0.0101 (6)	0.0003 (6)
C4A	0.0167 (7)	0.0123 (7)	0.0170 (7)	0.0019 (5)	0.0081 (6)	0.0004 (6)
C5A	0.0198 (8)	0.0158 (7)	0.0136 (7)	0.0021 (6)	0.0071 (6)	0.0005 (6)
C6A	0.0181 (8)	0.0151 (7)	0.0149 (7)	0.0012 (6)	0.0055 (6)	−0.0005 (6)
C7A	0.0149 (7)	0.0126 (7)	0.0156 (7)	0.0003 (5)	0.0058 (6)	0.0007 (6)
C8A	0.0150 (7)	0.0157 (7)	0.0165 (7)	−0.0015 (5)	0.0042 (6)	−0.0015 (6)
C9A	0.0145 (7)	0.0179 (8)	0.0183 (8)	−0.0027 (6)	0.0065 (6)	−0.0001 (6)
C10A	0.0149 (7)	0.0149 (7)	0.0154 (7)	0.0006 (5)	0.0063 (6)	0.0018 (6)
C11A	0.0136 (7)	0.0129 (7)	0.0148 (7)	0.0010 (5)	0.0057 (6)	0.0005 (6)
C12A	0.0148 (7)	0.0113 (7)	0.0143 (7)	0.0008 (5)	0.0065 (6)	−0.0002 (6)
C13A	0.0136 (7)	0.0162 (7)	0.0171 (7)	−0.0001 (5)	0.0070 (6)	−0.0006 (6)
C14A	0.0141 (7)	0.0182 (8)	0.0162 (7)	−0.0004 (5)	0.0066 (6)	0.0013 (6)
Cl1B	0.0236 (2)	0.0236 (2)	0.0259 (2)	−0.00425 (16)	0.01529 (18)	−0.00761 (17)
Cl2B	0.01594 (19)	0.0422 (3)	0.0212 (2)	0.01094 (18)	0.00581 (16)	0.0069 (2)
Cl3B	0.01827 (18)	0.01960 (19)	0.0235 (2)	−0.00159 (14)	0.00980 (16)	0.00239 (16)
Cl4B	0.0246 (2)	0.0411 (3)	0.0172 (2)	−0.01349 (19)	0.01067 (17)	−0.00318 (19)
Cl5B	0.01830 (19)	0.0254 (2)	0.0204 (2)	0.00703 (15)	0.00734 (16)	0.00478 (17)
Cl6B	0.0233 (2)	0.0233 (2)	0.01712 (19)	0.00652 (15)	0.00497 (16)	−0.00445 (16)
N1B	0.0153 (6)	0.0140 (6)	0.0156 (6)	0.0010 (5)	0.0067 (5)	0.0008 (5)
N2B	0.0145 (6)	0.0138 (6)	0.0152 (6)	0.0006 (5)	0.0068 (5)	−0.0012 (5)
C1B	0.0150 (7)	0.0141 (7)	0.0160 (7)	0.0011 (5)	0.0066 (6)	0.0013 (6)
C2B	0.0172 (8)	0.0176 (8)	0.0160 (7)	−0.0008 (6)	0.0053 (6)	0.0001 (6)
C3B	0.0204 (8)	0.0161 (8)	0.0139 (7)	0.0008 (6)	0.0064 (6)	−0.0006 (6)
C4B	0.0172 (7)	0.0121 (7)	0.0148 (7)	0.0016 (5)	0.0061 (6)	0.0016 (6)
C5B	0.0208 (8)	0.0166 (8)	0.0138 (7)	0.0020 (6)	0.0095 (6)	0.0021 (6)
C6B	0.0192 (8)	0.0164 (8)	0.0191 (8)	0.0018 (6)	0.0117 (7)	0.0026 (6)
C7B	0.0157 (7)	0.0118 (7)	0.0177 (7)	0.0005 (5)	0.0077 (6)	0.0002 (6)
C8B	0.0158 (7)	0.0161 (7)	0.0209 (8)	−0.0002 (5)	0.0105 (6)	−0.0001 (6)
C9B	0.0150 (7)	0.0173 (8)	0.0187 (8)	−0.0016 (6)	0.0068 (6)	−0.0020 (6)
C10B	0.0158 (7)	0.0138 (7)	0.0152 (7)	−0.0003 (5)	0.0074 (6)	−0.0017 (6)
C11B	0.0158 (7)	0.0111 (7)	0.0160 (7)	0.0005 (5)	0.0078 (6)	−0.0003 (6)
C12B	0.0149 (7)	0.0116 (7)	0.0154 (7)	0.0007 (5)	0.0070 (6)	0.0000 (6)
C13B	0.0127 (7)	0.0174 (7)	0.0161 (7)	0.0008 (5)	0.0045 (6)	0.0010 (6)
C14B	0.0124 (7)	0.0198 (8)	0.0145 (7)	−0.0002 (5)	0.0046 (6)	−0.0013 (6)

### Geometric parameters (Å, °)

**Table:**

Cl1A—C13A	1.7667 (18)	Cl1B—C13B	1.7690 (18)
Cl2A—C13A	1.7800 (17)	Cl2B—C13B	1.7746 (17)
Cl3A—C13A	1.7887 (18)	Cl3B—C13B	1.7870 (18)
Cl4A—C14A	1.7673 (18)	Cl4B—C14B	1.7625 (18)
Cl5A—C14A	1.7800 (17)	Cl5B—C14B	1.7834 (17)
Cl6A—C14A	1.7772 (18)	Cl6B—C14B	1.7839 (18)
N1A—C1A	1.319 (2)	N1B—C1B	1.319 (2)
N1A—C12A	1.354 (2)	N1B—C12B	1.352 (2)
N2A—C10A	1.318 (2)	N2B—C10B	1.319 (2)
N2A—C11A	1.353 (2)	N2B—C11B	1.355 (2)
C1A—C2A	1.406 (2)	C1B—C2B	1.403 (2)
C1A—C13A	1.529 (2)	C1B—C13B	1.529 (2)
C2A—C3A	1.372 (2)	C2B—C3B	1.379 (3)
C2A—H2AA	0.9300	C2B—H2BA	0.9300
C3A—C4A	1.405 (2)	C3B—C4B	1.408 (2)
C3A—H3AA	0.9300	C3B—H3BA	0.9300
C4A—C12A	1.413 (2)	C4B—C12B	1.416 (2)
C4A—C5A	1.434 (2)	C4B—C5B	1.434 (2)
C5A—C6A	1.356 (2)	C5B—C6B	1.354 (2)
C5A—H5AA	0.9300	C5B—H5BA	0.9300
C6A—C7A	1.430 (2)	C6B—C7B	1.429 (2)
C6A—H6AA	0.9300	C6B—H6BA	0.9300
C7A—C8A	1.408 (2)	C7B—C8B	1.409 (2)
C7A—C11A	1.420 (2)	C7B—C11B	1.415 (2)
C8A—C9A	1.368 (3)	C8B—C9B	1.373 (3)
C8A—H8AA	0.9300	C8B—H8BA	0.9300
C9A—C10A	1.408 (2)	C9B—C10B	1.408 (2)
C9A—H9AA	0.9300	C9B—H9BA	0.9300
C10A—C14A	1.533 (2)	C10B—C14B	1.526 (2)
C11A—C12A	1.461 (2)	C11B—C12B	1.457 (2)
			
C1A—N1A—C12A	117.40 (15)	C1B—N1B—C12B	117.69 (15)
C10A—N2A—C11A	117.80 (14)	C10B—N2B—C11B	117.80 (15)
N1A—C1A—C2A	124.57 (15)	N1B—C1B—C2B	124.64 (16)
N1A—C1A—C13A	115.06 (15)	N1B—C1B—C13B	114.72 (15)
C2A—C1A—C13A	120.35 (15)	C2B—C1B—C13B	120.63 (15)
C3A—C2A—C1A	117.74 (15)	C3B—C2B—C1B	117.71 (16)
C3A—C2A—H2AA	121.1	C3B—C2B—H2BA	121.1
C1A—C2A—H2AA	121.1	C1B—C2B—H2BA	121.1
C2A—C3A—C4A	119.90 (16)	C2B—C3B—C4B	119.67 (16)
C2A—C3A—H3AA	120.0	C2B—C3B—H3BA	120.2
C4A—C3A—H3AA	120.0	C4B—C3B—H3BA	120.2
C3A—C4A—C12A	117.41 (15)	C3B—C4B—C12B	117.55 (16)
C3A—C4A—C5A	121.86 (16)	C3B—C4B—C5B	121.71 (16)
C12A—C4A—C5A	120.72 (15)	C12B—C4B—C5B	120.73 (15)
C6A—C5A—C4A	120.61 (16)	C6B—C5B—C4B	120.36 (16)
C6A—C5A—H5AA	119.7	C6B—C5B—H5BA	119.8
C4A—C5A—H5AA	119.7	C4B—C5B—H5BA	119.8
C5A—C6A—C7A	120.75 (16)	C5B—C6B—C7B	121.01 (16)
C5A—C6A—H6AA	119.6	C5B—C6B—H6BA	119.5
C7A—C6A—H6AA	119.6	C7B—C6B—H6BA	119.5
C8A—C7A—C11A	117.04 (16)	C8B—C7B—C11B	117.17 (16)
C8A—C7A—C6A	122.40 (15)	C8B—C7B—C6B	122.41 (16)
C11A—C7A—C6A	120.55 (15)	C11B—C7B—C6B	120.41 (15)
C9A—C8A—C7A	120.16 (16)	C9B—C8B—C7B	120.10 (16)
C9A—C8A—H8AA	119.9	C9B—C8B—H8BA	119.9
C7A—C8A—H8AA	119.9	C7B—C8B—H8BA	119.9
C8A—C9A—C10A	117.89 (16)	C8B—C9B—C10B	117.73 (15)
C8A—C9A—H9AA	121.1	C8B—C9B—H9BA	121.1
C10A—C9A—H9AA	121.1	C10B—C9B—H9BA	121.1
N2A—C10A—C9A	124.26 (16)	N2B—C10B—C9B	124.30 (16)
N2A—C10A—C14A	114.98 (14)	N2B—C10B—C14B	115.15 (15)
C9A—C10A—C14A	120.75 (15)	C9B—C10B—C14B	120.54 (15)
N2A—C11A—C7A	122.63 (15)	N2B—C11B—C7B	122.70 (15)
N2A—C11A—C12A	118.78 (14)	N2B—C11B—C12B	118.42 (15)
C7A—C11A—C12A	118.57 (15)	C7B—C11B—C12B	118.86 (15)
N1A—C12A—C4A	122.83 (15)	N1B—C12B—C4B	122.58 (15)
N1A—C12A—C11A	118.56 (15)	N1B—C12B—C11B	118.99 (15)
C4A—C12A—C11A	118.60 (14)	C4B—C12B—C11B	118.42 (15)
C1A—C13A—Cl1A	111.92 (12)	C1B—C13B—Cl1B	111.59 (12)
C1A—C13A—Cl2A	111.35 (12)	C1B—C13B—Cl2B	111.42 (12)
Cl1A—C13A—Cl2A	107.65 (9)	Cl1B—C13B—Cl2B	108.11 (9)
C1A—C13A—Cl3A	109.01 (11)	C1B—C13B—Cl3B	109.25 (11)
Cl1A—C13A—Cl3A	108.73 (9)	Cl1B—C13B—Cl3B	108.67 (9)
Cl2A—C13A—Cl3A	108.08 (9)	Cl2B—C13B—Cl3B	107.68 (9)
C10A—C14A—Cl4A	111.35 (12)	C10B—C14B—Cl4B	112.10 (11)
C10A—C14A—Cl6A	109.72 (12)	C10B—C14B—Cl5B	110.87 (12)
Cl4A—C14A—Cl6A	108.72 (9)	Cl4B—C14B—Cl5B	107.66 (9)
C10A—C14A—Cl5A	111.23 (11)	C10B—C14B—Cl6B	109.13 (12)
Cl4A—C14A—Cl5A	107.56 (10)	Cl4B—C14B—Cl6B	108.79 (9)
Cl6A—C14A—Cl5A	108.16 (9)	Cl5B—C14B—Cl6B	108.17 (9)
			
C12A—N1A—C1A—C2A	−3.0 (2)	C12B—N1B—C1B—C2B	3.0 (2)
C12A—N1A—C1A—C13A	178.49 (14)	C12B—N1B—C1B—C13B	−178.33 (14)
N1A—C1A—C2A—C3A	3.8 (3)	N1B—C1B—C2B—C3B	−3.9 (3)
C13A—C1A—C2A—C3A	−177.75 (15)	C13B—C1B—C2B—C3B	177.48 (15)
C1A—C2A—C3A—C4A	−0.8 (3)	C1B—C2B—C3B—C4B	0.9 (3)
C2A—C3A—C4A—C12A	−2.4 (2)	C2B—C3B—C4B—C12B	2.5 (2)
C2A—C3A—C4A—C5A	176.67 (16)	C2B—C3B—C4B—C5B	−176.39 (16)
C3A—C4A—C5A—C6A	−178.26 (16)	C3B—C4B—C5B—C6B	177.95 (17)
C12A—C4A—C5A—C6A	0.8 (3)	C12B—C4B—C5B—C6B	−0.9 (3)
C4A—C5A—C6A—C7A	−2.6 (3)	C4B—C5B—C6B—C7B	3.2 (3)
C5A—C6A—C7A—C8A	−178.65 (17)	C5B—C6B—C7B—C8B	178.21 (17)
C5A—C6A—C7A—C11A	0.5 (3)	C5B—C6B—C7B—C11B	−1.2 (3)
C11A—C7A—C8A—C9A	−3.0 (2)	C11B—C7B—C8B—C9B	2.8 (2)
C6A—C7A—C8A—C9A	176.17 (16)	C6B—C7B—C8B—C9B	−176.64 (16)
C7A—C8A—C9A—C10A	−1.0 (3)	C7B—C8B—C9B—C10B	0.9 (3)
C11A—N2A—C10A—C9A	−2.9 (3)	C11B—N2B—C10B—C9B	3.1 (2)
C11A—N2A—C10A—C14A	178.02 (14)	C11B—N2B—C10B—C14B	−178.51 (14)
C8A—C9A—C10A—N2A	4.2 (3)	C8B—C9B—C10B—N2B	−4.1 (3)
C8A—C9A—C10A—C14A	−176.80 (16)	C8B—C9B—C10B—C14B	177.53 (16)
C10A—N2A—C11A—C7A	−1.5 (2)	C10B—N2B—C11B—C7B	1.2 (2)
C10A—N2A—C11A—C12A	−179.67 (15)	C10B—N2B—C11B—C12B	179.64 (15)
C8A—C7A—C11A—N2A	4.4 (2)	C8B—C7B—C11B—N2B	−4.0 (2)
C6A—C7A—C11A—N2A	−174.77 (15)	C6B—C7B—C11B—N2B	175.45 (15)
C8A—C7A—C11A—C12A	−177.43 (15)	C8B—C7B—C11B—C12B	177.49 (15)
C6A—C7A—C11A—C12A	3.4 (2)	C6B—C7B—C11B—C12B	−3.0 (2)
C1A—N1A—C12A—C4A	−0.7 (2)	C1B—N1B—C12B—C4B	0.9 (2)
C1A—N1A—C12A—C11A	−179.56 (15)	C1B—N1B—C12B—C11B	179.52 (15)
C3A—C4A—C12A—N1A	3.3 (2)	C3B—C4B—C12B—N1B	−3.6 (2)
C5A—C4A—C12A—N1A	−175.76 (15)	C5B—C4B—C12B—N1B	175.35 (15)
C3A—C4A—C12A—C11A	−177.82 (15)	C3B—C4B—C12B—C11B	177.77 (15)
C5A—C4A—C12A—C11A	3.1 (2)	C5B—C4B—C12B—C11B	−3.3 (2)
N2A—C11A—C12A—N1A	−8.0 (2)	N2B—C11B—C12B—N1B	7.9 (2)
C7A—C11A—C12A—N1A	173.80 (15)	C7B—C11B—C12B—N1B	−173.53 (15)
N2A—C11A—C12A—C4A	173.16 (15)	N2B—C11B—C12B—C4B	−173.35 (15)
C7A—C11A—C12A—C4A	−5.1 (2)	C7B—C11B—C12B—C4B	5.2 (2)
N1A—C1A—C13A—Cl1A	−30.18 (18)	N1B—C1B—C13B—Cl1B	34.79 (18)
C2A—C1A—C13A—Cl1A	151.19 (14)	C2B—C1B—C13B—Cl1B	−146.50 (14)
N1A—C1A—C13A—Cl2A	−150.73 (13)	N1B—C1B—C13B—Cl2B	155.75 (13)
C2A—C1A—C13A—Cl2A	30.6 (2)	C2B—C1B—C13B—Cl2B	−25.5 (2)
N1A—C1A—C13A—Cl3A	90.12 (16)	N1B—C1B—C13B—Cl3B	−85.40 (16)
C2A—C1A—C13A—Cl3A	−88.50 (17)	C2B—C1B—C13B—Cl3B	93.31 (17)
N2A—C10A—C14A—Cl4A	−33.57 (19)	N2B—C10B—C14B—Cl4B	27.50 (19)
C9A—C10A—C14A—Cl4A	147.36 (14)	C9B—C10B—C14B—Cl4B	−154.01 (14)
N2A—C10A—C14A—Cl6A	86.84 (16)	N2B—C10B—C14B—Cl5B	147.86 (13)
C9A—C10A—C14A—Cl6A	−92.23 (17)	C9B—C10B—C14B—Cl5B	−33.7 (2)
N2A—C10A—C14A—Cl5A	−153.53 (13)	N2B—C10B—C14B—Cl6B	−93.09 (15)
C9A—C10A—C14A—Cl5A	27.4 (2)	C9B—C10B—C14B—Cl6B	85.40 (17)
